# High-Efficiency Small Sample Microparticle Fractionation on a Femtosecond Laser-Machined Microfluidic Disc

**DOI:** 10.3390/mi11020151

**Published:** 2020-01-30

**Authors:** Ala’aldeen Al-Halhouli, Zaid Doofesh, Ahmed Albagdady, Andreas Dietzel

**Affiliations:** 1 NanoLab, School of Applied Technical Sciences, German Jordanian University (GJU), Amman 11180, Jordandoofesh@gmail.com (Z.D.); ahmed.albagdady@gju.edu.jo (A.A.); 2 Institut für Mikrotechnik, Technische Universität Braunschweig, 38124 Braunschweig, Germany; 3 Faculty of Engineering, Middle East University, Amman 11831, Jordan

**Keywords:** microfluidics, femtosecond laser, microparticle separation, microfluidic disc

## Abstract

The fabrication and testing of microfluidic spinning compact discs with embedded trapezoidal microchambers for the purpose of inertial microparticle focusing is reported in this article. Microparticle focusing channels require small features that cannot be easily fabricated in acrylic sheets and are complicated to realize in glass by traditional lithography techniques; therefore, the fabrication of microfluidic discs with femtosecond laser ablation is reported for the first time in this paper. It could be demonstrated that high-efficiency inertial focusing of 5 and 10 µm particles is achieved in a channel with trapezoidal microchambers regardless of the direction of disc rotation, which correlates to the dominance of inertial forces over Coriolis forces. To achieve the highest throughput possible, the suspension concentration was increased from 0.001% (w/v) to 0.005% (w/v). The focusing efficiency was 98.7% for the 10 µm particles and 93.75% for the 5 µm particles.

## 1. Introduction

Particle/cell separation of biological samples is a crucial step in sample preparation for various biomedical assays. It has received more interest, since it is a key tool for the study of individual cells or particles [1,2] that has already enabled great discoveries in cell biology and precise patient health diagnosis [3,4]. It has also been applied to many other applications, including separation of magnetic particles from a background mixture [5,6]. Compared to traditional separation methods, microfluidics is proven to have improved recovery and purity rates [7]. Microfluidics has also enabled combining multiple parallel lab procedures onto a single chip, which greatly improves flexibility and portability. Moreover, the introduction of microfluidics has enabled miniaturization of previously larger systems and lead to the development of cheap and disposable platforms to purify and concentrate samples. An advantage of microfluidic platforms is the reduced volume of raw materials needed to operate while allowing accuracy, low-price, compactness, and high throughput.

The flow inside microchannels can be induced in two main methods; one is by using an external pump, such as a syringe pump or an high performance liquid chromatography (HPLC) pump [8], while another is utilizing centrifugal forces in a disc-shaped platform. In such a design, microfluidic features, known as “chambers”, contain the sample fluid, while other features, known as “channels”, connect different chambers and act as a passage for fluids inside the disc. A special spinning system setup rotates the disc and subjects the fluid to a centrifugal force that is sufficient for fluid migration, resulting in a unidirectional flow away from the disc’s center. The external pump method is more commonly used due to its ability to introduce precise and accurate flow rates; however, using a microfluidic disc requires no tubes or connections, and it is a simple method that has a huge potential in automated testing and parallelization [9,10]. Liquid control is usually more challenging in microfluidic discs because the design is rotating and it is not easy to insert valves or sensors in the channels; however, many research groups have tried to solve many of the challenges addressed. Passive valving methods are used to control fluids in microchannels by changing the channel geometry, as in capillary valves [11]. Depending on the channel’s material, capillary valves behave differently. Hydrophobic valves create high surface tension forces that oppose the flow, which can be performed by either coating the channel with a hydrophobic material or by reducing the channel’s width (flow constriction). On the other hand, fluid flow in hydrophilic channels (glass) is quite the opposite, because fluids flow passively inside the channel and stop at a geometry expansion if the surface tension force is higher than the centrifugal force caused by the rotating disc [12]. The main disadvantages of passive methods is that only one input parameter can be accessed, which is the disc rotational speed, and it hugely depends on the disc’s material whether it is hydrophobic or hydrophilic. It also gets harder when increasing the number of valves in the disc as the valve’s burst speed is usually not sharp and changes from experiment to experiment if the channel’s wetting condition changed. Active methods provide more flexibility and repeatability than passive methods and they are less sensitive to the disc’s material; however, they require an external power source and might be complicated to fabricate and actuate. Some active methods use a destructible physical barrier to block the fluid flow [13], such as wax [9] and ice plugs [14], while other methods utilize reusable techniques, such as thermo-pneumatic valving [15] and magnetic valving [13]. Several applications have been developed based on microfluidic discs, such as immunoassays [16,17], cell lysis [18], the prothrombin time test [19], micro-mixing [20], and valving [9,21]. Spinning systems can be categorized as self-sufficient pumping systems that drive fluids from one chamber to another, executing specific reactions in a predefined order [22]. Amongst other self-sufficient systems, rotational systems provide more control to the user, resulting in a possibility of creating repeatable complex experiments by only varying the rotational speed of the microfluidic disc; however, in simpler low-pressure applications, it might be cheaper to use other passive techniques, such as capillary action [23] and paper microfluidics [24]. Other recent interesting techniques included using commercially available latex balloons as pressure pumps when connected to a syringe [25]. Such inexpensive and disposable setups are proven to perform hydrodynamic cells manipulation, large monocytes capturing, and rapid solution exchange [26].

Inertial microparticle separation showed various advantages, such as the ability to process large sample volumes with high throughput and high recovery rate, together with the ability of multiplexing for improved performance, which is in contrast to active methods, such as dielectrophoretic [27] and magnetic separation [28]. Passive methods in general do not require an external force to isolate particles; therefore, implementing inertial separation on microfluidic discs should be very simple to operate. Such a method has proven to be a simple and efficient way to produce high purity blood plasma [29]. However, this method lacks the opposing force needed to fractionate particles based on properties as size, and it depends on the rotation direction of the disc. Other techniques have been developed for the purpose of implementing inertial forces in a microfluidic rotational flow with an integrated micromixer for creating a microbead-cancer cell complex [30]. In traditional inertial separation methods based on pump-driven flow, inertial lift and drag forces mainly control the equilibrium position of microparticles. The inertial lift force ($F_{L}$) is the resultant of the wall-lift and shear-gradient lift forces that act in opposing directions and in different magnitudes, depending on flow velocity, particle diameter, fluid density, and channel geometry. On the other hand, Dean drag force ($F_{D}$) is introduced in curved channels and leads to migration of smaller particles laterally towards the outer side wall of curvature. New forces are introduced in rotational flow systems, such as Coriolis and centrifugal forces that can change the velocity profile inside the channel; hence changing particles equilibrium positions. Integrating trapezoidal microchambers along the channel’s side wall is proven to benefit the focusing efficiency in terms of the focusing position and focusing line’s width [31]. In this work, we study microfluidic discs with straight channels and integrated trapezoidal microchambers as a method for low sample isolation of 5 and 10 μm particles, resulting in a focusing efficiency of ~92% for 5 μm particles and ~98% for 10 μm particles. Femtosecond laser ablation is used as the fabrication method to produce smooth, hydrophilic, dimensionally stable, and precise microchannels. A comparison between different microfluidic disc fabrication materials is also discussed to emphasize the advantage of selecting the glass material. 

## 2. Materials and Methods

### 2.1. Custom Made Portable Spinning System

**Figure 1:**
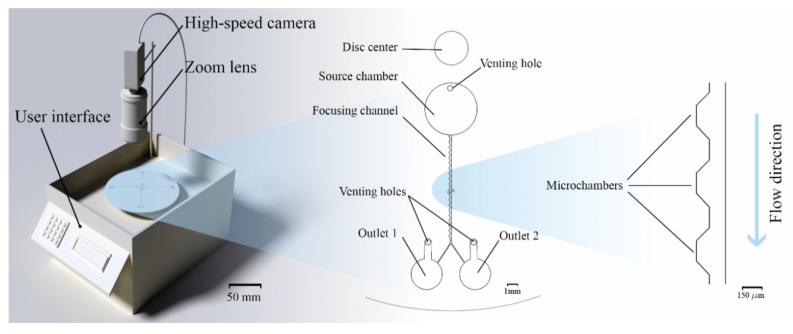
Three-dimensional (3D) illustration of the spinning system with all its components, and the microfluidic design of the focusing channel. Microchambers are implemented along one side of the focusing channel to enhance particle focusing.

Part of this work describes the development of an accurate and reliable spinning system based on the concept followed in a previous work [20], but with modifications in software and hardware interfaces. The new system (check Figure 1) is smaller in size and operable as standalone and portable. Another upgrade made to the system was implementing the FLIR Grasshopper GS3-U3-23S6C-C camera (FLIR systems, Wilsonville, OR, USA) with superior quantum efficiency (74% at 525 nm) and short minimum exposure time (5 µs), enabling us to capture images of a spinning disc at speeds as high as 4000 rpm without motion blur. Finally, the spinning system comprises a blue light-emitting diode (LED) illumination array to excite the green fluorescence of microparticles visible in the camera. 

### 2.2. Glass vs Polymethyl Methacrylate (PMMA) vs Polydimethylsiloxane (PDMS)

Acrylic glass or PMMA (Polymethyl methacrylate) is widely used in microfluidic disc fabrication [15,32,33] because they are biocompatible, affordable, and easy to cut and engrave by CO_2_ laser cutters or computer numerical control (CNC) machines. The fabrication process using PMMA usually includes engraving multiple acrylic layers to create channels and other microfluidic features and then carefully placing a pressure sensitive adhesive (PSA) layer between every two PMMA layers for bonding [34,35]. Once all layers are bonded with PSA, the disc is placed in a hydraulic press for a few minutes to increase layer adhesion and prevent any possible leakage. Precise alignment is required in this method as it becomes difficult in complex-multilayer designs. Additionally, acrylic is more prone to scratches compared to glass, and the additional adhesion layers may result in a decrease in optical transparency that is crucial for experiment observation. PMMA sheets have a higher optical transparency; however, glass discs can be made much thinner that PMMA sheets with the same thickness are flexible and might cause vibration when rotating on a spinning system. A summary of different microfluidic disc manufacturing processes were discussed in a work by Gilmore et al. [10], where fabrication techniques, such as polymer molding, CNC machining, and the print-cut-lamination (PCL) method were compared in terms of affordability, disposability, and fabrication material. Machining glass wafers with a femtosecond laser workstation is an expensive process, primarily due to the cost of the machine itself, as well as the subsequent bonding process in a regulated clean environment; however, in an application such as inertial separation, the microparticle equilibrium position is highly sensitive to the channel geometry, and any undesired obstacle that may have been the result of fabrication may disrupt particle’s trajectory. Additionally, microparticle focusing usually requires small channel dimensions to provide enough hydrodynamic forces. Fabrication of microfluidic discs from glass wafers is not very common to the community, since it requires a more complicated and time-consuming process, but it can engrave channels as small as 30 μm in width and results in superior fabrication accuracy and provides systems with higher pressure endurance.

It is also possible to create a hybrid Polydimethylsiloxane (PDMS)-PMMA microfluidic disc by spin coating PDMS on top of a PMMA layer that acts as a support for the microfluidic design. PDMS is very popular in the microfluidic community due to its low fabrication costs, simple setup, and fast prototyping capability [36,37,38]. In spite of the PDMS being biocompatible, optically transparent, non-flammable, and exhibiting a good thermal and chemical stability [36,39], it has many drawbacks, such as the mechanical softness limiting the structure’s aspect ratio, the swelling when exposed to various organic solvents, and the tendency to absorb drug proteins and small hydrophobic molecules. But mainly in microfluidics and inertial separation, where high pressure values occur in the microchannels, PDMS, with its low modulus of elasticity, limits achievable flow rates, because a rupture in the chip [40,41] or a difference between the expected and actual flow rates [40] can occur. 

Glass is known for its dimensional stability, multi-layer design capability, thermal resistance, and chemical inertness [36,37,38,42]. Glass fabrication can be performed by lithography, combined with wet chemical etching or plasma etching, or laser ablation. However, glass fabrication can cost more than PMMA and PDMS as it requires access to femtosecond laser machining and a cleanroom.

### 2.3. Microfluidic Disc Design

Different inertial microfluidic designs on microfluidic discs are fabricated and tested to focus fluorescent microparticles. As shown in Figure 1, the design contains one 5 mm×240 μm (diamter×depth) circular source chamber and two 3 mm×240 μm destination chambers, connected by a separation channel. Four different designs were fabricated in one disc to allow for parallel testing. In principle, more than 20 channels could be realized in the case of a single disc-center-concentric source chamber. A design implemented in an earlier work [31] about integrating microchambers along spiral microfluidic channels was considered and returned promising results that will be discussed in a following section. The main focusing channels has a width of 120 µm and a depth of 50 µm (the drawing of the focusing channel is provided in the Supplementary Material Drawing S1). Images of the spinning system setup and the fabricated glass microfluidic disc are available in the Supplementary Material Figures S1–S7.

**Figure 2:**
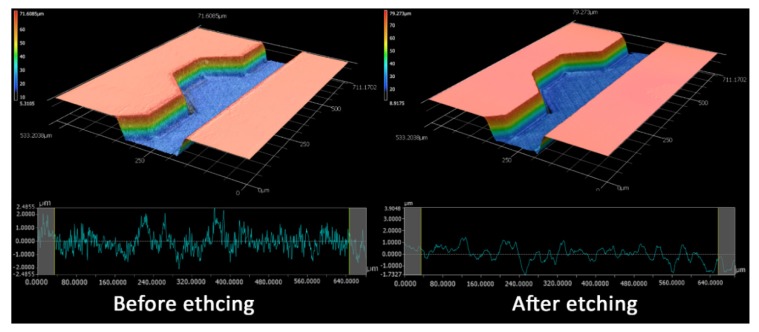
A 3D reconstruction of the fabricated channel showing the difference in surface roughness before and after the glass etching process. The arithmetic average values for the roughness profile ($R_{a}$) are 0.6755 µm and 0.4571 µm before and after glass etching, respectively.

The microfluidic channels were drafted in AutoCAD 2018 software (Autodesk, San Rafael, CA, USA) and were fabricated in a femtosecond-laser workstation (microSTRUCT-C, 3D-Micromac, Germany) using laser strategies described in a previous work [43]. The channels and chambers were engraved using a fill strategy, which consists of 4 sets of intersecting lines that cover the area to be ablated and represents the tracks the laser will pass. Contour lines were added to straighten the side walls, since the channel resulting from the filling step only would have inclined side walls. Glass wafers used were 0.7 mm thick and 100 mm in diameter (BOROFLOAT^®^, Schott AG, Mainz, Germany). After laser ablation, the wafer was processed in a clean environment and steeped in a glass etching solution (Phosphoric acid, Hydrofluoric acid and water, 20:6:9) for 90 s in order to smoothen the ablation facets and dissolve residual glass fragments. The ablated wafer and another blank wafer were then inserted in a wafer cleaning machine (Fairchild Convac, Neuenstadt, Germany) that sprays pressurized water and dispenses a mixture of H_2_SO_4_ and H_2_O_2_ for cleaning and surface activation before thermally bonding the two wafers by placing them in a muffle oven at 620° for a duration of 6 h. The post-ablation process was adopted from an earlier work by Erfle et al. [44,45]. Figure 2 shows laser microscopy images before and after dipping the wafer in glass etching solution. Both the ablation facets and the not-structured wafer surface appeared to be smoother and without undesired glass fragments, which resulted from laser ablation. The roughness is an important feature as it prevented particles from being trapped inside the cavities of the channel, especially when working with particles of 5 µm or smaller. 

### 2.4. Fractionation Mechanism

**Figure 3:**
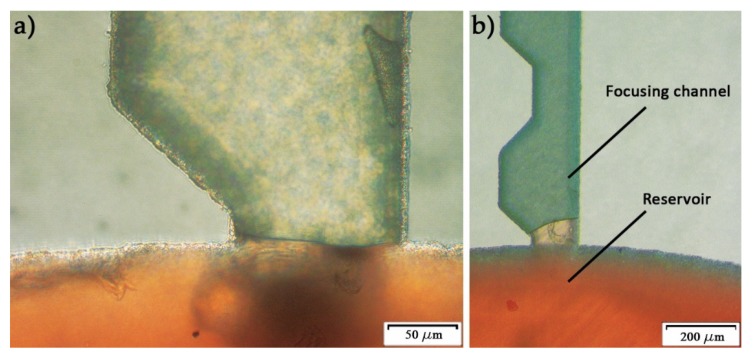
Dyed deionized (DI) water flow experiment inside the focusing channel. Picture (**a**) shows the fluid stopping at the channel inlet due to the pressure barrier caused by the width expansion, while picture (**b**) is taken after rotating the microfluidic disc at 1500 rpm.

For centrifugal microfluidic fractionation, two kinds of forces need to be established; one drives the fluid and results in the velocity profile, while another affects the microparticles and promotes particle migration. First of all, it is important to understand that even though microparticle focusing in centrifugal and pump-based setups share the same purpose, they are very different in application, because centrifugal microfluidic platforms are limited to the prefilled volume; therefore, they are more suited for diagnostics and rapid test applications. As a result, the flowrate is not directly controlled in the channels, and the motor rotational speed usually replaces the flow rate as the primary control parameter. Usually in glass capillary channels, the fluid flows passively due to the high hydrophilicity of the glass surface; however, the particle solution is found to never leave the source chamber when the disc is at rest. Figure 3a shows red dyed deionized (DI) water that filled the source chamber (reservoir) and stopped at the beginning of the focusing channel. Even after rotating the disc at a rotational speed up to 1500 rpm, yet the water didn’t go pass the first microchamber inlet, as seen in Figure 3b. The flat meniscus in hydrophilic surfaces indicates the presence of back pressure inside the focusing channel due to high fluidic resistance in the small channels. To test this hypothesis, one outlet was sealed, while the other was connected to a tube, and suction was applied to reduce the pressure in the channel, simulating a channel with low fluidic resistance. The meniscus became concave instantly after the suction was applied, as can be seen in the Electronic Supplementary Material Video S1. Conventionally in plastic systems, capillary force opposes the centrifugal force because the channel surface is hydrophobic; however, in glass systems, the only force opposing the centrifugal force is the force generated by the back pressure inside the microchannels. Therefore, the term “burst frequency”, which usually represents the rotational speed at which the centrifugal force is slightly higher than the capillary force, causing the fluid to leave the source chamber, would in this work correlate to the balance with the back pressure instead of the capillary pressure.

There are other forces acting on fluids in microfluidic discs, such as the Coriolis and Euler forces, which act in a direction perpendicular to the direction of the fluid flowing in the channel. Euler force is proportional to the angular acceleration [46]; therefore, it is expected to be at the highest when the motor starts and vanishes when the disc is rotating at a constant speed. On the other hand, Coriolis force depends on the angular velocity magnitude and is expected to be higher at higher rotational speeds. These forces are given by the formula:

(1) $$F_{cor}=\;-2m\omega \times \frac{dr}{dt}\;$$

(2) $$F_{Eul}=-m\frac{d\omega }{dt}\times r{\;}_{\;}$$

where m is the fluid element mass with a position of r from the centre of rotation and an angular velocity of ω. 

As a result of the above-mentioned forces affecting the fluid, suspended microparticles experience inertial forces in different directions and magnitudes depending on the shape of the fluid velocity profile, fluid properties, particle size, and other physical parameters. The generated asymmetric wake when a particle is close the wall directs the particles away from the wall with a force known as the wall lift force ($F_{LW}$) [47]. The typical parabolic velocity profile in a microchannel causes an opposing shear-gradient lift force ($F_{LS}$), which is effective in high shear areas close to the channel walls. The resultant of these two forces is known as lift force [48].

(3) $$F_{L}=\frac{2\rho U_{f}{}^{2}a_{c}{}^{4}}{D_{h}}\;$$

where $a_{c}$ is the particle diameter, $U_{f}$ is the average fluid velocity, ρ is the fluid density, and $D_{h}$ is the hydraulic diameter. In case of a curved channel, a secondary flow develops that affects particle position by redistributing the velocity profile [49,50]. The two counter-rotating vortices that appear above and below the center line of the channel’s cross section are known as Dean vortices, which induce the Dean force ($F_{D}$) acting on the particle. The strength of the Dean vortices can be represented by the dimensionless number $D_{e}$ as: [51]

(4) $$D_{e}=Re\sqrt{\frac{D_{h}}{2R}}\;$$

where Re is the Reynolds number and R is the radius of curvature. The particle migration velocity is called the Dean velocity and is expressed as $U_{Dean}=1.8\times 10^{-4}De^{1.63},\;$and $F_{D}$ can be written as:

(5) $$F_{D}=3\pi \mu U_{Dean}a_{c}$$

The channel must provide a minimum length for particles to ensure full lateral migration and spiral channels can contain a long channel in a small area due to its continuous curvature. However, microfluidic discs are unidirectional flow devices; hence it is not passively possible to curve the channel towards the disc’s center and maintain a liquid flow. Therefore, the maximum possible channel length of 30 mm was utilized in this work.

**Figure 4:**
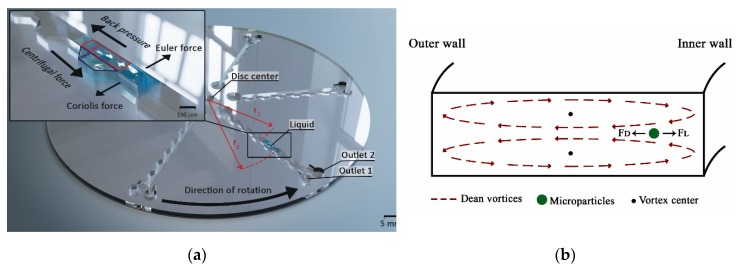
(**a**) 3D representation of the design with an illustration of forces affecting a fluid element passing through the channel. The channel size is exaggerated for demonstration purposes. (**b**) A cross-section in a rectangular spiral microchannel showing the direction of Dean vortices and the forces affecting microparticles flowing in that channel.

In a previous work [31], microchambers were implemented to shift the particles from the center of a spiral channel to the inner wall by changing the velocity profile between consecutive microchambers. Different shapes of microchambers were tested and evaluated with fluorescent 2 µm particles to explore suitable designs in terms of focusing the line’s width and distance from the inner wall. Microchamber designs included inclined microchamber walls forming a trapezoidal shape that prevents particles from overshooting as they exit. A continuously curved channel is impossible to realize in a microfluidic spinning disc, because after the first quadrant, the fluid direction will oppose the centrifugal force and the flow will stop. Another method to induce a Dean flow without needing to reverse the flow direction, with respect to the centrifugal force, is by using an asymmetric curving system on a microfluidic chip, as was described by Di Carlo et al. [52] and implemented on a microfluidic disc by Aguirre et al. and Kitsara et al. [30,53]. Contraction-expansion arrays (CEAs) [54] can generate a secondary flow, because the fluid streamlines follow a curved path as they enter the expansion region, which creates a pressure gradient that results in a Dean flow. The designs evaluated in this work consider microchambers on one side of a straight microchannel to focus particles towards the microchamber side independently from the disc rotation direction. Figure 4a shows a 3-dimensional illustration of the focusing channel in the microfluidic disc with the forces affecting a fluid column. This flow in a spinning disc system is very complex to analyze [55], because a pressure gradient can arise from Coriolis force as well as the difference in fluid velocity between areas A and B in Figure 4a. However, the same equations used in pump-driven systems can be applied to rotational systems given the same flow conditions, with the rotational speed replacing the flow rate term. The fluid velocity, particle diameter, and the channel geometry are the major parameters that govern the particle equilibrium position, as seen in Equations (1) and (2). In general, smaller channel dimensions, bigger particle diameter, and higher flow rate result in higher forces and increase the possibility of successful particle focusing; however, since most of the particle diameter is a given constant parameter, the design must include a specific channel geometry and an optimized flow rate to achieve particle focusing. The inertial lift and Dean drag forces are represented in Figure 4b, showing the different directions of lift and Dean forces acting on a microparticle.

### 2.5. Particle Suspension Preparation

Polystyrene fluorescent particles (exc./emm.: 468/508 nm, 1% solids concentration, Fluoro-Max, Thermo Fisher Scientific, Massachusetts, USA) of sizes 5 and 10 µm were used. In each experiment, two concentrations were tested; 50 µL of particle solution were mixed with 10 mL of DI water in a vortex mixer, resulting in concentrations of 0.005% w/v, while 10 µl of particle solution with 10 mL DI water produced a 0.001% w/v suspension concentration.

## 3. Results and Discussion

**Figure 5:**
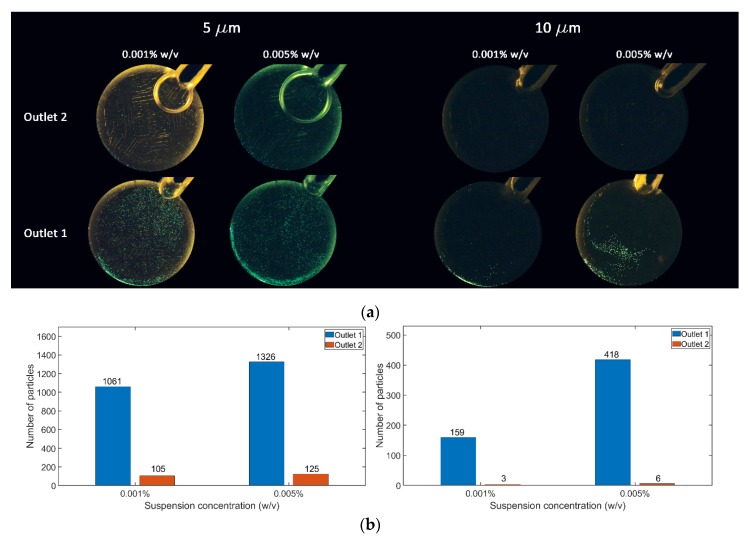
5 and 10 µm particles focusing results at 0.001% (w/v) and 0.005% (w/v) suspension concentrations. (**a**) Outlet chambers after particle fractionation experiments. (**b**) The effect of particle suspension concentration on focusing efficiency for 5 µm particles (left) and 10 µm particles (right). Results show that higher concentrations return higher throughput while approximately maintaining the same focusing efficiency.

After fixing a microfluidic disc on the spinning system platform and injecting the particle solution into the source chamber, the disc’s rotational speed must exceed the burst frequency so that the solution can flow in the focusing channel. Experimentally, the burst frequency was found to be 1800 rpm. High rotational speeds cause particle sedimentation in the destination chamber, making the quantification process harder; therefore, particle focusing was observed on 2000 and 4000 rpm to find the lowest speed at which focusing can be achieved. Suspension concentration is often overlooked as an essential parameter of focusing efficiency, however, along with the focusing flowrate, it directly correlates with the channel’s throughput. Using higher concentrations may result in multiple equilibrium positions for the same particle size [56]. Figure 5a shows the experimental results on the design with trapezoidal microchambers on one side of the focusing channel, 5 µm particles was focused at 4000 rpm, while 10 µm was focused at 2000 rpm, because lift forces depend greatly on the particle diameter, as discussed earlier. There was no difference after changing the direction of rotation in these experiments, which means that Coriolis force had little to no effect on particle focusing, and since 5 and 10 µm particles have $a_{c}/D_{h}\geq 0.07$, then lift forces are dominant and are responsible for particle focusing in the channel [52,57]. The trapezoidal microchambers contribute in shifting the focusing position towards them by gradual entry and exit, as optimized in a previous study [31]. The results were monitored after the experiment on an inverted microscope (Leica DM IL LED) equipped with a blue LED light source (Leica SFL 100, both Leica Microsystems GmbH, Wetzlar, Germany). The focusing efficiency was calculated optically by processing the destination chamber images in a MATLAB script that counts the bright spots in an input image given the approximate spot diameter. This method was sufficient to capture the trend of focusing efficiency. Figure 5b shows the particle count in the destination chambers near and opposite to microchambers (outlet 1 and outlet 2 respectively) after fractionation experiments under different suspension concentrations. For the 10 um particles, the count was on average 418 particles in outlet 1 and 6 particles in outlet 2, resulting in a 98.58% focusing efficiency at 0.005% (w/v) concentration. On the other hand, a focusing efficiency of 91.39% was achieved for the 5 µm particles. Increasing the rotational speed of the microfluidic disc to achieve higher efficiency for smaller particles might not be the best approach, since glass microfluidic discs are brittle and might break when subjected to high centrifugal force; furthermore, higher flow rates causes the emergence of a second velocity maximum point, causing multiple particle focusing positions [58], while, in other cases, increasing the flow rate doesn’t cause a change in the particle equilibrium position. A new design with smaller features would be a good approach to specifically target particles smaller than 5 µm in diameter. Using microfluidic discs for inertial focusing is a promising rapid technique for small sample processing as it takes a few seconds for the fluid to pass through the focusing channel to the destination chambers. Minimal system complexity and usability are great features of the proposed method, since spinning systems are easy to operate and do not required tubes and connectors as in conventional pump-driven systems.

## 4. Conclusions

Fabricating microfluidic discs with femtosecond laser ablation is a promising technique as it requires less steps than soft lithography and achieves repeatable results with the inert glass material. Pump-driven systems for low volume samples can be extremely complicated and unsuitable for particle fractionation. Therefore, a precise spinning system was developed, which is not costly and can be built in any lab. The camera and illumination systems are optional and results can be monitored with any inverted microscope with a blue light. Coriolis force has no effect on particle focusing in the suggested design, since no difference was observed when changing the disc’s direction of rotation, which means lift forces were responsible for particle lateral migration. Trapezoidal microchambers were embedded on one side of the focusing channel to shift the equilibrium position toward the side wall. This behavior occurred because the side wall got gradually further as a particle entered a microchamber; therefore, the wall lift force vanished, causing the particle to migrate inside the microchamber. As a result, inertial focusing with 98.58% efficiency for 10 µm particles and 91.39% for 5 µm particles was achieved while using different suspension concentrations of 0.005% (w/v) and 0.001% (w/v). 

## Supplementary Materials

The following are available online at https://www.mdpi.com/2072-666X/11/2/151/s1, Drawing S1: AutoCAD design of the microfluidic disc, Figures S1–S7: pictures of the spinning system and glass microfluidic disc, Video S1: Meniscus change after suction is applied at the outlet.
